# Serious Overestimation in Quantitative PCR by Circular (Supercoiled) Plasmid Standard: Microalgal *pcna* as the Model Gene

**DOI:** 10.1371/journal.pone.0009545

**Published:** 2010-03-05

**Authors:** Yubo Hou, Huan Zhang, Lilibeth Miranda, Senjie Lin

**Affiliations:** Department of Marine Sciences, University of Connecticut, Groton, Connecticut, United States of America; University of Canterbury, New Zealand

## Abstract

Quantitative real-time PCR (qPCR) has become a gold standard for the quantification of nucleic acids and microorganism abundances, in which plasmid DNA carrying the target genes are most commonly used as the standard. A recent study showed that supercoiled circular confirmation of DNA appeared to suppress PCR amplification. However, to what extent to which different structural types of DNA (circular versus linear) used as the standard may affect the quantification accuracy has not been evaluated. In this study, we quantitatively compared qPCR accuracies based on circular plasmid (mostly in supercoiled form) and linear DNA standards (linearized plasmid DNA or PCR amplicons), using proliferating cell nuclear gene (*pcna*), the ubiquitous eukaryotic gene, in five marine microalgae as a model gene. We observed that PCR using circular plasmids as template gave 2.65-4.38 more of the threshold cycle number than did equimolar linear standards. While the documented genome sequence of the diatom *Thalassiosira pseudonana* shows a single copy of *pcna*, qPCR using the circular plasmid as standard yielded an estimate of 7.77 copies of *pcna* per genome whereas that using the linear standard gave 1.02 copies per genome. We conclude that circular plasmid DNA is unsuitable as a standard, and linear DNA should be used instead, in absolute qPCR. The serious overestimation by the circular plasmid standard is likely due to the undetected lower efficiency of its amplification in the early stage of PCR when the supercoiled plasmid is the dominant template.

## Introduction

Quantitative real-time polymerase chain reaction (qPCR) is a powerful technique that allows accurate and sensitive quantification of starting amounts of DNA without post-PCR manipulation [1]. QPCR in combination with reverse transcription (qRT-PCR) is rapidly becoming the method of choice for mRNA (converted to cDNA) quantification, and is often recommended for the validation of microarray data [2], [3], [4]. It is also an essential technique for quantifying gene (or noncoding DNA) copy number in a cell [5], [6]. Real-time PCR quantification methods are broadly classified as “relative” or “absolute” [7]. Relative qPCR measures the differences in abundances of the target DNA or RNA (reverse-transcribed to cDNA) between samples without showing their actual abundances, and the comparison can only be done for samples run within the same qPCR reaction. Absolute qPCR allows the precise quantification of the target DNA/cDNA based on a standard curve constructed in the same quantification assay as the question samples. The standard curve in an absolute qPCR is generated by amplifying a dilution series of a standard DNA, which can be a plasmid (including phagemid) DNA carrying the target DNA, a PCR amplicon, a synthesized oligonucleotide, a genomic DNA, or a cDNA. Among the various types of standard DNA, plasmid DNA, especially the uncut circular one, is the most common choice due to its high stability and reproducibility. It has been shown that uncut circular plasmid DNA is mostly in supercoiled form [8], and that the supercoiled structure of the untreated template plasmid DNA can suppress real-time PCR compared to other relaxed templates [9]. It has also been suggested that careful discrimination of quantitative changes due to either copy number change or structural disruption is needed [9], and linearization may need to be considered for a plasmid to be used as a standard in qPCR (http://www.appliedbiosystems.com/support/tutorials/pdf/quant_pcr.pdf). However, the magnitude of error a circular plasmid standard may cause and what other conformational types of DNA can be a better choice of standard remain obscure.

In this study, we evaluated three most common forms of standard DNA: circular plasmid, linearized plasmid (digested by restriction enzyme), and linear PCR amplicon. Proliferating cell nuclear antigen gene (*pcna*), a ubiquitous gene in eukaryotes, from four dinoflagellates and a diatom was used as the model gene for the study. Quantification accuracies of real-time PCR assays based on different standards were compared. Consistently, significant differences were observed in the threshold cycle number (Ct) between the circular plasmid and linear (linearized plasmid or linear PCR amplicon) DNA. We further used these different conformational types of DNA as standard in qPCR to quantify the *pcna* copy number in the fully sequenced *T. pseudonana* genome. Our results demonstrated that the linear DNA standards including linearized plasmids, but not the circular plasmid standard, were reliable for absolute qPCR.

## Methods

### Microalgal Cultures

The monoclonal cultures of four harmful bloom-forming dinoflagellates and one fully sequenced diatom were used in this study. The dinoflagellate *Alexandrium fundyense* CA28 was provided by D. M. Anderson at Woods Hole Oceanographic Institution. The dinoflagellates *Karlodinium veneficum* CCMP1975, *Prorocenrum micans* CCMP1589, and *Prorocentrum minimum* CCMP696, and the diatom *T. pseudonana* CCMP1335 were obtained from the Provasoli-Guillard National Center for Culture of Marine Phytoplankton (CCMP, West Boothbay Harbor, Maine). *A. fundyense* was grown in F/2-Si seawater medium at 15°C under a 14:10 h light: dark cycle. *K. veneficum*, *P. micans*, and *P. minimum* were grown in F/2-Si seawater medium at 20°C under a 12:12 h light: dark cycle. *T. pseudonana* was grown in F/2 seawater medium at 15°C under a 14:10 h light: dark cycle. All algal cultures were grown under a photon flux density of 100 µE m^−2^ s^−1^. Cell concentrations were measured in triplicate using Sedgwick-Rafter counting chambers.

### DNA and RNA Extraction and cDNA Library Construction

Microalgal cell samples were harvested by centrifugation at 4°C under 3000×g for 20 min. The *A. fundyense* and *P. micans* cell pellets were homogenized using a micropestle to break the theca on the cell surface before nucleic acid extractions as reported [10]. Other species used in this study had weak theca and hence the homogenization step was omitted. For DNA extraction, the cell pellet of each species was resuspended and incubated overnight in 500 µl of DNA extraction buffer (10 mM Tris-HCl, 100 mM EDTA, 0.5% w/v sodium dodecyl sulfate, pH = 8.0) with 200 µg ml^−1^ proteinase K. Genomic DNA (gDNA) was extracted using a CTAB (cetyltrimethylammonium bromide) protocol [11]. After extraction with chloroform, gDNA was further purified using the Zymo DNA Clean and Concentrator kit (Zymo Research, Orange, California) to remove any remaining impurities. GDNA was finally dissolved in 10 mM Tris-HCl buffer (pH = 8) and stored at −20°C. GDNA concentration was measured using a NanoDrop ND-1000 spectrophotometer (Thermo Scientific, Wilmington, Delaware). For RNA extraction, the cell pellet was resuspended in1 ml of Trizol Reagent (Invitrogen, Carlsbad, California) and stored in -80°C if not processed immediately. Total RNA was isolated as reported [12]. Alternatively, RNA was extracted using RNAeasy Mini kit (Qiagen, Valencia, California). RNA was dissolved in Diethylpyrocarbonate (DEPC)-treated water and stored at −80°C. The full-length cDNA of *K. veneficum* was obtained previously [13]. The first-strand cDNA of other algae was synthesized using GeneRacer kit following manufacturer instruction (Invitrogen, Carlsbad, California).

### PCR-Based Cloning and Sequencing of *pcna* cDNA

Proliferating cell nuclear antigen gene (*pcna*) was chosen as the model in this study because it is a common gene in all eukaryotes and it is a target of our research as a potential cell cycle marker for algal growth rate studies [14]. For *T. pseudonana*, a *pcna* fragment was amplified from its gDNA using the specific primer set TpspcnaF1-TpspcnaR1 designed based on its *pcna* sequence shown in the recently released genome sequence (http://genome.jgi-psf.org/Thaps3/Thaps3.home.html). For *A. fundyense*, *P. micans* and *P. minimum*, *pcna* fragments were amplified using the first strand cDNA as the template and the spliced leader-based primer (DinoSL) paired with DinoPCNA3d as the primer set (Table 1) under the condition previously reported [14]. For *K. veneficum*, a *pcna* fragment was amplified from its full-length cDNA using DinoPCNA5c-RACER3′. PCR amplicon was purified using the Zymo DNA Clean and Concentrator kit and cloned into pBluescript II KS vectors (2963 bp, Stratagene, La Jolla, California) using Takara DNA Ligation kit v.1 (TakaraBioUSA, Madison, Wisconsin). Clones were randomly picked and plasmid DNA was isolated from 2 ml of bacterial culture using the Qiaprep Spin Miniprep kit to avoid the contamination by bacterial RNA that may occur with a non-column-based plasmid isolation method. *Pcna* insert was sequenced using the BigDye Terminator Cycle Sequencing kit (Applied Biosystems, Foster City, California). Plasmid DNA was dissolved, measured, and stored in the same way as gDNA described above.

**Table 1:** PCR primers used in this study.

Primer name	Primer sequence (5′→3′)	PCR annealing temperature (°C)
**Regular PCR**		
DinoSL^a^	TCC GTA GCC ATT TTG GCT CAA G	55
DinoPCNA3d^b^	TCG TCG ATC TTS GGN GCN AGR TAR AA	
DinoPCNA5c^b^	ATC GCC GGA CTT YGA RCT NAA RCT NAT G	55
RACER3′^c^	GCT GTC AAC GAT ACG CTA CGT AAC G	
PmicpcnaF2	GCG TTC TCT GAG TTC AAG TGT GAC	60
PmicpcnaR	GCT CGT GGA CTG TGA GGG TC	
TpspcnaF1	GCA AGC ACG CCT CAC CCA AG	60
TpspcnaR1	CTC ATC CTT CTC CGC AGC ACT ATT C	
**QPCR**		
***Alexandrium fundyense***		
AfupcnaF	CAG GTG AAG GCA AGC AAG GA	57
AfupcnaR	GTT GTC AGT CTT CTC AAG GTC YTA C	
***Karlodinium veneficum***		
KvepcnaF	GGA GAT GTY GGH ACW GGN AAT GT	56.5
KvepcnaR	TAG AAY TGC ATG TAD CCR TTG TC	
***Prorocentrum micans***		
PmicpcnaF1	GAG CAG CAV TAC AAG GTG GTG G	60
PmicpcnaR	GCT CGT GGA CTG TGA GGG TC	
***Prorocentrum minimum***		
PminpcnaF	ATH GAG AGC GAG CAC ATG GAG	65
PminpcnaR	GCT CCA CSG TKC CGC ACA G	
***Thalassiosira pseudonana***		
TpspcnaF2	GAC CTA GTC CAA GAA GCC AAC ATA G	66-60 touch-down
TpspcnaR2	AAC ACC AAC GCC AAC GAA TCC	

a Zhang et al. 2006, 2007.

b Zhang et al. 2006.

c GeneRacer kit, Invitrogen, Carlsbad, California.

### Construction of Circular Plasmid and Linear DNA Standards

Circular plasmid and linear standards were compared to examine the effect of DNA structural confirmation on PCR result and amplification efficiency. Linearized plasmid DNA (3592-3816 bp) and PCR amplicon (436–866 bp) were compared to examine the effects of length and source of DNA (bacterial or PCR amplified). In the *A. fundyense* qPCR, the *pcna* recombinant plasmid DNA prepared as mentioned above was used as the circular plasmid standard, named **AfuC1** (3816 bp) (Table 2). In order to minimize the experimental error and test the plasmid purity, a second circular plasmid standard (**AfuC2**) was prepared by further purifying **AfuC1** using the Zymo DNA Clean and Concentrator kit. Two linearized plasmid standards for *A. fundyense*, **AfuL1** and **AfuL2**, were prepared by digesting **AfuC1** with restriction endonuclease EcoRI (4 bp away from the *pcna* insert) and SalI (26 bp away from the *pcna* insert), respectively. In parallel, a linear PCR amplicon standard for *A. fundyense*, **AfuL3** (853 bp), was prepared by amplifying the *pcna* fragment using **AfuC1** as the template and DinoSL-DinoPCNA3d as the primer set. Similarly, the circular plasmid standards for *P. micans* (**PmicC**), *P minimum* (**PminC**), *K. veneficum* (**KveC**), and *T. pseudonana* (**TpsC**) (3592–3829 bp) were prepared as for **AfuC1**. The linearized plasmid standards for *K. veneficum* (**KveL**) were similarly prepared as for **AfuL1**. The linear PCR amplicon standards of *P. micans* and *T. pseudonana* (**PmicL** and **TpsL** respectively) were generated from the respective gDNA. The linear PCR amplicon standard of *P. minimum* (**PminL**) was amplified from PminC. The complete linearization of the circular plasmid was confirmed by checking the band pattern in the agarose gel. All PCR amplicon standards were purified using the Zymo DNA Clean and Concentrator kit. The optical absorbance at OD_260_ was measured in triplicates using NanoDrop ND-1000 spectrophotometer. Based on the OD_260_ value and the DNA sequence, the molar concentration of the standard DNA was calculated using the OligCalc oligonucleotide properties calculator [15], and then converted into copy number of DNA molecules per unit volume (in the order of magnitudes of 10^9^–10^11^ copies µl^−1^). The standard was finally prepared in dilution series (1×10^2^ to 1×10^6^–10^7^ copies µl^−1^) for qPCR. Standard DNA was freshly prepared before use to avoid degradation that may occur during storage.

**Table 2:** Types and performance of standard DNA in qPCR in various algal species examined.

Algal species	Standard name	Standard type^a^	Length (bp)	Standard curve^b^ (R^2^)	E (%)^c^
***Alexandrium fundyense***	**AfuC1**	Circular plasmid bearing EF133957	3816	y = −3.642x+40.152 (1.000)	88.2
	**AfuC2**	Circular plasmid bearing EF133957	3816	y = −3.673x+39.770 (0.997)	87.2
	**AfuL1**	Linearized plasmid bearing EF133957	3816	y = −3.799x+36.790 (1.000)	83.3
	**AfuL2**	Linearized plasmid bearing EF133957	3816	y = −3.707x+36.376 (0.998)	86.1
	**AfuL3**	PCR amplicon based on DinoSL-DinoPCNA3d primer set	853	y = −3.477x+35.471 (1.000)	93.9
***Karlodinium veneficum***	**KveC**	Circular plasmid bearing partial EF134029	3592	y = −3.888x+44.484 (0.999)	80.8
	**KveL**	Linearized plasmid bearing partial EF134029	3592	y = −3.897x+40.237 (0.999)	80.6
***Prorocentrum micans***	**PmicC**	Circular plasmid bearing EF133939	3820	y = −3.420x+40.272 (1.000)	96.1
	**PmicL**	PCR amplicon based on PmicpcnaF2-PmicpcnaR primer set	436	y = −3.523x+38.059 (1.000)	92.2
***Prorocentrum minimum***	**PminC**	Circular plasmid bearing EF134019	3829	y = −3.679x+40.874 (0.999)	87.0
	**PminL**	PCR amplicon based on DinoSL-DinoPCNA3d primer set	866	y = −3.834x+37.560 (1.000)	82.3
***Thalassiosira pseudonana***	**TpsC**	Circular plasmid bearing gene fragment bounded by primer set TpspcnaF1-TpspcnaR1	3631	y = −3.921x+45.462 (0.999)	79.9
	**TpsL**	PCR amplicon based on TpspcnaF1-TpspcnaR1 primer set	668	y = −4.029x+42.437 (0.997)	77.1

a. The plasmid vector is pBluescript II KS (2963 bp).

b. The linear regression equation between Ct (y) and log_10_ starting copy number (x).

c. Efficiency calculated as E = (10^(−1/slope)^ -1)×100%.

### Microalgal *pcna* qPCR Assays

Five algal *pcna* qPCR were carried out on iCycler iQ Real-Time PCR detection system with SYBR Green supermix (Bio-Rad, Hercules, California). *Pcna*-specific qPCR primers were designed for each species using the program Beacon Designer (Table 1). The specificity of the primers was verified by analyzing the qPCR melt curve and sequencing the PCR amplicon. The standard DNA was diluted in 5–6 serial steps and applied in duplicate (2×10^2^ to 2×10^6^–10^7^ copies per reaction). In the case of *T. pseudonana*, three gDNA samples were used as the target DNA, each in six dilutions (100 pg, 200 pg, 500 pg, 1 ng, 2 ng, and 4 ng per reaction) and each dilution was applied in triplicate, which allowed comprehensive evaluation of PCR efficiency and quantification accuracy across a broad range of target DNA quantities. The qPCR condition included a single denaturation cycle of 95°C for 3 min, 40 cycles of 95°C for 20 s, annealing at primer-specific temperature for 30 s (Table 1), and elongation at 72°C for 15 sec.

### Analyses of Threshold Cycle, Amplification Efficiency, and Genomic *pcna* Copy Number

The threshold cycle number (Ct) was reported by the iCycler iQ program under the “PCR baseline subtracted” option. The standard curve was generated as linear regression between Ct and log_10_ starting copy number of standard DNA. The iQ program automatically calculated the amplification efficiency (E) of the standard DNA from the slope of the standard curve: E = 10^(−1/slope)^-1. Based on a statistical model in a previous study (Equation 5 in [16]), a multiple regression model was built using SPSS 15 to test the slope and Ct differences between equimolar circular and linear DNA in each qPCR. The model was y = a+b_1_x_1_+b_2_x_2_+b_3_ x_1*_x_2_+ε, where the dependent variable (y) is threshold cycle (Ct), the covariate (x_1_) is the logarithmic-transformed known *pcna* copy number in the standard DNA, the fix factor (x_2_) is the DNA type (circular or linear, coded as 0 or 1 by SPSS 15), and ε is the error. If the coefficient of the interaction term (b_3_) is significant (*p*<0.05), the slopes of the standard curves (and hence amplification efficiencies) for the circular and the linear standards are significantly different from each other. If the coefficient of the fix factor (b_2_) is significant (*p*<0.05), Ct values for the two types of standards are significantly different. The differences in Ct values (ΔCt) were calculated as the average Ct difference across serial dilutions. When the slopes of the two standard curves (or efficiencies) are significantly different, Ct difference was adjusted (ΔCt') as the average of (Ct_1_*E_1_′-Ct_2_*E_2_′) across all dilution levels, where E' is another form of amplification efficiency commonly used and also calculated from the slope: E' = log_2_10^(−1/slope)^ (modified from equation 7 in [17]).

In the case of *T. pseudonana* qPCR, the starting *pcna* copy number in each dilution of *T. pseudonana* gDNA sample (i.e., qPCR-estimated *pcna* copy number) was calculated based on TpsC and TpsL, respectively. The qPCR-estimated copy number was compared with the expected number calculated according to 1 *pcna* per haploid genome (34 Mbp or 0.035 pg of gDNA) [18] (http://genome.jgi-psf.org/Thaps3/Thaps3.home.html). We also did the linear regression analysis between Ct and log_10_ amount of *T. pseudonana* gDNA. From the slope of the regression line, the amplification efficiency of *T. pseudonana* gDNA was calculated as E = 10^(−1/slope)^-1.

## Results

### Remarkably Different Ct Values for Circular Plasmid and Linear Standards

**Figure 1:**
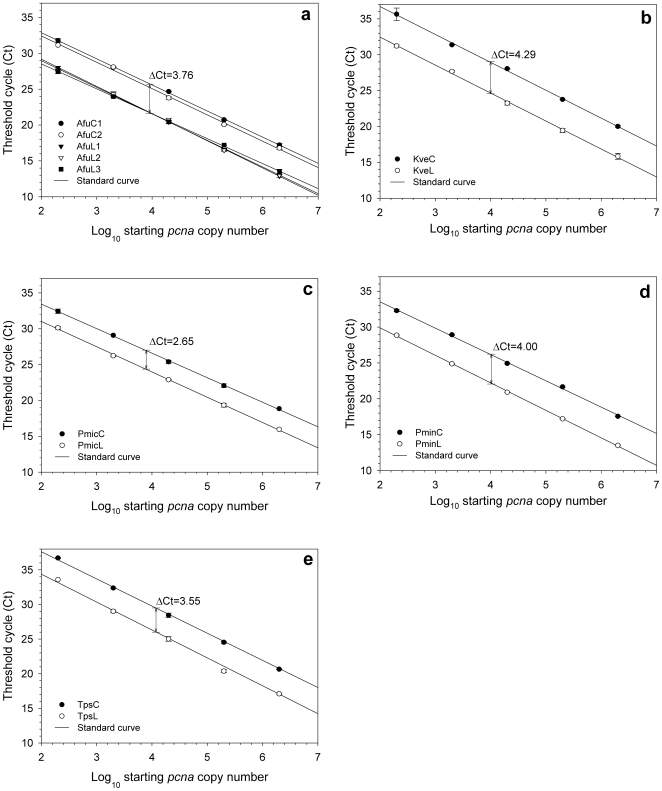
Standard curves with similar slopes and significant threshold cycle differences (ΔCt) between circular and linear standards in the *pcna* qPCR for (a) *Alexandrium fundyense*, (b) *Karlodinium veneficum*, (c) *Prorocentrum micans*, (d) *P. minimum*, and (e) *Thalassiosira pseudonana*. Standard curves were linear regression lines between Ct and Log_10_ starting *pcna* copy number (calculated from standard DNA concentration), each based on a type of standard DNA. Note that similar slopes of the standard curves indicate similar amplification efficiencies. All ΔCt were calculated as the average Ct difference across serial dilutions and statistically significant (*p*<0.001). The error bars denote the standard deviations of Ct values among replicates.

The threshold cycle numbers (Ct) of circular plasmid standards ranged from 16.79 to 36.72, and Ct of the linearized plasmid and PCR amplicon standards (collectively named linear standards) ranged from 12.89 to 33.59. In all cases, the circular plasmid and the equimolar linear DNA had significant different Ct values (*p*<0.001). In the *A. fundyense* qPCR, the circular plasmid prepared by the Qiaprep Spin Miniprep kit (AfuC1) and the other further purified by the DNA Clean and Concentrator kit (AfuC2) yielded similar Ct values without significant difference (ΔCt = 0.53). For the linearized plasmid standards (AfuL1-2, 3816 bp) and the PCR amplicon (AfuL3, 853 bp), despite the differences in length, very small ΔCt (0.33) was observed. In contrast, remarkable Ct differences were observed between circular plasmid and linear standards (*p*<0.001, ΔCt = 2.65–4.29) (Fig. 1a-e). As shown in Fig. 1a, we found that the mean Ct of AfuC1-2 were markedly higher than those of AfuL1-3 (*p*<0.001, ΔCt = 3.76). Consistently, in all other *pcna* qPCR experiments, the Ct values of the circular plasmid standards were higher than the linear ones: ΔCt = 4.29 in *K. veneficum*, ΔCt = 2.65 in *P. micans*, ΔCt = 4.00 in *P. minimum*, and ΔCt = 3.54 in *T. pseudonana* (*p*<0.001 in all cases). The conformational state of qPCR standard DNA appeared to exert strong influence on their Ct, with substantially higher values from the circular plasmid than linear standards.

### Comparison of Amplification Efficiencies between Circular Plasmid and Linear DNA Standards

All standard curves were generated with high coefficients of determination (R^2^ = 0.998–1.000) (Table 2). In each qPCR, different standard curves appeared parallel (Fig. 1a–e) and thus the efficiencies derived from their slopes were similar. In the *A. fundyense* qPCR, the overall coefficient of variation (or CV, equal to the standard deviation divided by the mean) of the efficiencies for all five *A. fundyense* standards was 4.45%. The efficiencies between circular plasmids (AfuC1-2) and linear DNA (AfuL1-3) were not significantly different (*p* = 0.977), neither for the two circular (*p* = 0.724) or linearized (*p* = 0.231) plasmids. However, among AfuL1-3, the CV of efficiencies was slightly increased (6.13%), and the efficiency for AfuL3 was significantly different from the other two (*p*<0.001). In the *K. veneficum*, *P. micans*, and *T. pseudonana* qPCR, efficiencies for the circular plasmid and linear DNA were highly similar (*p* = 0.934, 0.197, and 0.387 and CV = 0.18%, 2.93%, and 2.52%, respectively). In *P. minimum*, the efficiencies for PminC and PminL were significantly different although the difference was fairly small (*p* = 0.011 and CV = 3.93%). Incorporating the efficiency difference between PminC and PminL, the Ct difference after adjustment (ΔCt' = 4.38) was slightly larger than that without adjustment (ΔCt = 4.00). In summary, no consistent difference in amplification efficiencies was observed between circular and linear standard DNA.

### *Pcna* Copy Number in *T. pseudonana* gDNA

**Figure 2:**
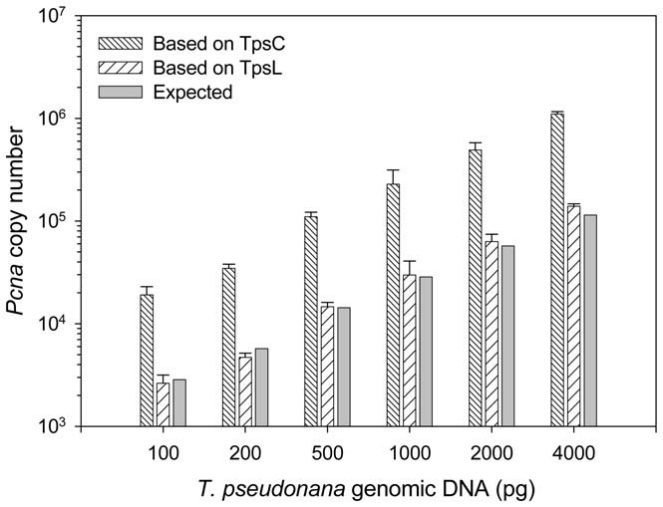
Comparison of qPCR-estimated and expected *pcna* copy numbers in *Thalassiosira pseudonana* gDNA samples. The expected copy numbers were calculated based on 1 *pcna* per genome (0.035 pg of gDNA). Note that the copy number estimates based on the linear standard (TpsL) are similar with the expected numbers, while those based on the circular standard (TpsC) are much higher than the expected values. The error bars denote the standard deviations.

Significantly different copies of *pcna* were estimated based on TpsC and TpsL (*p*<0.001) for each *T. pseudonana* gDNA dilution sample (100 pg to 4000 pg per reaction) (Fig. 2). The copy numbers measured from TpsL were very similar to the expected numbers calculated according to 1 copy of *pcna* per the 0.035 pg DNA genome of *T. pseudonana*, whereas the estimates from the circular TpsC were 7.77±1.28 times higher (n = 6). Using the results from the TpsL standard, a *T. pseudonana* haploid genome (0.032 pg of DNA) was estimated to contain 1.02±0.14 copies of *pcna* (n = 3), in close agreement with the actual 1 copy per genome value. In stark contrast, using supercoiled circular standard resulted in an estimate of 7.77±1.28 copies per genome (n = 3), indicative of serious overestimation by this standard (Table 3).

**Table 3:** QPCR-estimated and expected *pcna* gene copy numbers (mean ± standard deviation) in *Thalassiosira pseudonana* genomic DNA samples based on the circular (TpsC) and linear (TpsL) qPCR standards.

gDNA (n = 3)	100 pg	200 pg	500 pg	1000 pg	2000 pg	4000 pg	Estimated/expected ratio (n = 6)
**Based on TpsC (circular)**	19098±3862	34556±3437	110044±11644	228333±85468	490222±88525	1098222±61153	7.77±1.28
**Based on TpsL (linear)**	2633±531	4707±454	14627±1513	29867±10926	63200±11200	139267±7580	1.02±0.14
**Expected** ^a^	2857	5714	14286	28571	57143	114286	-

a. Calculated based on 1 *pcna* per 0.035 pg of gDNA.

## Discussion

Plasmid DNA containing the target sequence has been commonly used as the standards in quantitative real-time PCR due to its high stability (i.e. little degradation during storage) and ease in preparation [7]. In most applications, circular (i.e. undigested) plasmid gene clones are used directly without linearization, and little attention has been paid to the possible effect of conformational state on quantification accuracy. Other types of standard DNA may be utilized in rare cases, but an explanation for selecting the standard DNA is usually not provided (e.g., PCR amplicon standard used in [19]). In this study, we have conducted a systematic evaluation on the most common types of standards and demonstrated that the linearized plasmid or linear PCR amplicon is the type of choice for a qPCR standard. Our result showed that at any concentration applied (2×10^2^–2×10^7^copies per reaction), the circular plasmid DNA increased Ct by 2.5 more cycles compared to the linear DNA, and in accordance the standard prepared from the circular plasmid DNA led to 8-fold overestimation of *T. pseudonana pcna* copy number whereas the linear standards gave highly accurate estimates.

Differences observed between the different types of standards were not due to variations in our experiment operation or quality of template DNAs. In this study, the standard DNA and genomic DNA were carefully and freshly prepared to minimize artifacts and each DNA was used in replicated dilution series to assess the intra-assay variations. The gDNA extraction method (CTAB method combined with DNA binding column purification step) has been proved to be effective in removing potential PCR inhibitors rich in algal species [11]. Because the DNA quantification in this study relied on the optical absorbance, the gDNA extraction solvents did not include any phenol which potentially influences OD_260_ and overestimates the nucleic acid concentration. The silica column-based plasmid DNA extraction procedure also avoided the contamination by proteins and PCR inhibitory compounds. In the *T. pseudonana pcna* qPCR assay, the genomic *pcna* copy number was calculated based on copy number per unit DNA instead of per cell to avoid the potential effect of cell loss or variable gDNA extraction efficiency on the accuracy. In addition, the comparison between the two standards was always made using the same gDNA samples. The highly consistent results from the multiple qPCR assays and the low variances among the replicates clearly indicate that the qPCR assays in this study were robust, highly reproducible and the evaluation results are reliable.

Why did the circular plasmid standard result in significantly greater threshold cycle number (Ct) in qPCR than the linear standards? It has been noted that uncut circular plasmid DNA is mostly in supercoiled form [8]. For instance, 92% of pBluescript II KS and several other plasmids purified using the standard alkaline lysis and ethanol precipitation method was in supercoiled form [20]. According to the manufacturer information, the QIAprep miniprep kit used in this study also results in mostly supercoiled plasmid (http://www1.qiagen.com/Plasmid/AgaroseGelAnalysis.aspx). If nicks are introduced at opposite positions on both plasmid DNA strands, e.g., by restriction enzyme digestion, a plasmid is linearized and the supercoiling is relaxed. There is evidence that PCR is suppressed by supercoiling of the template DNA, and that the relaxing of DNA supercoil structure could increase the efficiency for primer binding and elongation in a PCR reaction [9]. This explains well the higher Ct values for circular plasmid than that for linearized plasmid. However, by multiple linear regression analyses, we did not find efficiency differences between the circular and linear DNA in all qPCR that can account for the differential Ct values. Only in one case did we observe a small difference in efficiency, which however contradicted rather than accounted for the Ct difference. It seems likely that the difference in Ct values and quantification accuracies lie in the first several cycles of qPCR when the supercoiled plasmid is the dominant template. Previous research has shown that the efficiency difference in the first few cycles would result in dramatic different qPCR results [21], such as ΔCt measured in this case. However, the efficiencies calculated from the standard curves do not reflect the differences in the early amplification stage, because the standard curves were constructed based on the Ct values identified in the exponential amplification stage (varied from 12.89 to 36.72 in this study) when linear PCR amplicon has become dominant and quantitatively outcompletes the supercoiled plasmid for amplification. Even if the amplification efficiency were calculated using such other methods as one using fluorescent data collected during PCR [22], [23], the initial lower efficiency of the supercoiled plasmid DNA still may not be easily detected.

While qPCR results from undigested plasmid DNA standard are strikingly different from those based on linear standards, linearized plasmid and linear PCR amplicon provide similar quantifications. This suggests that the length and source of the DNA template does not have significant effect on PCR efficiency. Practically each of these linear standards has its own advantage and the choice depends on convenience. Plasmid is stable for long-term storage and linearization can be carried out easily at time of standard preparation. PCR amplicon standard comes with the flexibility that it can be amplified from the stored plasmid if already available or directly from the genomic DNA of the target organism bypassing the tedious gene cloning procedure. Although our observations were based on *pcna* in marine microalgae, the findings likely apply to other genes and other organisms, because all the qPCR reactions are run under in vitro conditions. The only possible exception would be when the target DNA itself is circular (especially if it is in supercoiled state), such as uncut mitochondrial, viral, bacterial, or plasmid DNA, in which whether linear standard still gives more accurate result needs to be individually investigated. In light of our findings in this study, previous results of qPCR based on circular plasmid standards need to be revisited.
